# Microbiological behavior of 3D printing materials for indirect restorations: A scoping review

**DOI:** 10.4317/jced.63656

**Published:** 2026-01-28

**Authors:** Pedro Thiago de Oliveira Neves, Jade Véras Diniz, Laura Buarque Caminha Lins, Jéssica Marcela de Luna Gomes, Bruno Gustavo da Silva Casado, Rafaella de Souza Leão

**Affiliations:** 1 Master’s student, Division of Oral Rehabilitation, Faculty of Dentistry, University of Pernambuco (UPE), Recife, PE, Brazil; 2 Doctor of Dental Surgery, Faculty of Dentistry, University of Pernambuco (UPE), Recife, PE, Brazil; 3 Phd student, Division of Oral Rehabilitation, Faculty of Dentistry, University of Pernambuco (UPE), Recife, PE, Brazil; 4 Assistant Professor, Department of Dental Materials and Prosthodontics, Araçatuba Dental School, São Paulo State University (UNESP), Araçatuba, SP, Brazil; 5 Adjunct Professor, Division of Oral Rehabilitation, Faculty of Dentistry, University of Pernambuco (UPE), Recife, PE, Brazil; 6 Adjunct Professor, Division of Oral Rehabilitation, Faculty of Dentistry, University of Pernambuco (UPE), Recife, PE, Brazil

## Abstract

**Background:**

Although 3D-printed indirect restorations offer precision and reduced fabrication time, the literature still lacks consensus regarding their surface characteristics and microbiological behavior, factors that may reduce restoration longevity, reinforcing the need to consolidate the available evidence. The purpose of this scoping review was to map the available evidence on the microbiological behavior of 3D printing materials for indirect restorations.

**Material and Methods:**

The scoping review was conducted according to the guidelines of Arksey and O'Malley and the Joanna Briggs Institute, following the PRISMA-ScR checklist. The literature search was performed in PubMed, Scopus, and Web of Science databases, including in vitro, in vivo, in situ, and clinical studies addressing this topic.

**Results:**

Among the 20 included studies, 19 were conducted in vitro and only one in vivo. The main factors identified as reducing microbial adhesion were the incorporation of nanoparticles (such as ZrO2, TiO2, graphene, and silanized chitosan), appropriate surface polishing, and controlled post-curing, which decreased surface roughness and enhanced antimicrobial properties. Conversely, the absence of surface finishing, insufficient post-curing time, and certain polymer compositions were associated with increased bacterial adhesion.

**Conclusions:**

Both material modification and post-fabrication treatment are key determinants of the microbiological behavior of 3D-printed resins. Furthermore, factors such as printing parameters and finishing and polishing protocols have a direct influence on the microbiological performance of these materials.

## Introduction

Understanding the microbiological aspects of restorative materials is of paramount importance, as the oral cavity represents a complex environment where various species of the resident microbiota can colonize restorations, teeth, oral mucosa, and periodontal tissues in a pathogenic manner (1 , 2). Biofilm formation and bacterial adhesion are closely associated with several material-related factors involved in the fabrication of indirect restorations, including 3D-printed materials. Therefore, alterations in surface properties, such as roughness, hydrophobicity, and chemical composition, can influence the microbiological profile (3 , 4). In this context, the development of novel 3D-printed materials with antibacterial properties has garnered increasing attention in recent literature, intending to create restorative materials that contribute to the prevention of diseases such as dental caries and periodontitis (5). In the pursuit of improved microbiological performance, various resin-based materials have been developed and evaluated (6). Nanohybrid and nanoparticle-filled composites are regarded as universal restorative materials due to their favorable physical and esthetic properties (7). Some studies have explored the incorporation of fillers into 3D-printed resin matrices to enhance key attributes such as wear resistance and antimicrobial effectiveness (8 , 9). Furthermore, hybrid 3D-printed resins reinforced with ceramic particles have demonstrated enhanced physical and biological performance, suggesting promising applicability in long-term crown restorations (5 , 10 , 11). In addition to intrinsic properties of restorative materials, the continuous evolution of 3D printing technology introduces printing parameters that may influence both the microbiological behavior and the material characteristics of printed resins. Printing parameters, including printing type and orientation, significantly influence the final properties of these materials, particularly their mechanical and surface characteristics (12). Despite the growing body of research, the literature still lacks consensus regarding the microbiological behavior of 3D-printed materials. While some studies have reported minimal microbial adhesion and satisfactory surface smoothness (1 , 13 , 14), others have found increased surface irregularities and greater microbial colonization (2 , 11 , 15). Therefore, the present study aimed to map the available evidence regarding the microbiological behavior of 3D-printed materials used in indirect restorations. The null hypothesis was that 3D-printed restorative materials would not differ from those fabricated by other methods regarding microbial adhesion.

## Material and Methods

This scoping review was structured based on the five-stage methodological framework proposed by Arksey and O'Malley (16), which includes: identifying the research question; identifying relevant studies; selecting studies; charting the data; and collating, summarizing, and reporting the results. The review was also guided by the Joanna Briggs Institute Manual for Evidence Synthesis (17) and the Preferred Reporting Items for Systematic Reviews and Meta-Analyses extension for Scoping Reviews (PRISMA-ScR) (18). The protocol for this review was registered on the Open Science Framework platform (DOI: 10.17605/OSF.IO/KWQZV). The research was guided by the following question: "What is the microbiological behavior of 3D-printed materials used for indirect restorations?" Based on this, the Population Concept Context (PCC) framework was applied. The population was defined as materials used for the fabrication of indirect restorations; the concept referred to microbiological behavior; and the context was established as the digital workflow in dentistry. An electronic search was conducted across three databases: PubMed/MEDLINE, Web of Science, and Scopus. An initial search strategy was developed and subsequently adapted for each database. The search terms were organized into three groups: #1 (Dental Materials OR Acrylic Resins OR Provisional Restorations OR Composites OR Dental Polymers OR crown OR crowns OR Surface properties); #2 (3D printed OR 3-dimensionally printed OR Additive manufacturing OR Printing, Three-Dimensional OR 3D printing technology OR 3D printing OR CAD/CAM OR three-dimensional printed resin OR Computer-aided design OR Computer-aided manufacturing); #3 (Microbial adhesion OR Streptococcus OR Candida albicans OR Microbial response OR Biofilm formation OR Microorganisms OR Anti-Biofilm Formation OR Bacterial adhesion OR Streptococcus mutans OR Antimicrobial activity OR Microbiological behavior OR Streptococcus sanguinis OR Lactobacillus salivarius); The final search strategy applied was: #1 AND #2 AND #3. An electronic search was conducted up to February 2025. The search was independently performed by two reviewers (P.T.O.N., J.V.D.). Eligibility criteria were applied to select the studies based on evaluating titles and abstracts using the EndNote reference manager (EndNote; Clarivate), and studies that did not meet these criteria were excluded. In the subsequent phase, the complete texts of all potentially eligible studies were examined by the same calibrated reviewers. In cases where there was no consensus among the researchers, a third (R.S.L.) was consulted. One researcher (P.T.O.N.) piloted the extraction form on a few studies and extracted the following data from the articles: author, year of publication, study type, test material, control material, evaluated microbiological aspects, additional parameters assessed, and study conclusions, using data extraction tables (Excel Microsoft corporation). In case of missing data, the authors of the included studies were contacted via email to provide the missing or additional data. Another researcher (J.V.D.) reviewed the data. The inclusion criteria comprised in vitro studies, in vivo studies, clinical studies, and in situ investigations that evaluated the microbiological behavior of materials and used 3D-printed materials. Exclusion criteria were defined as follows: case-reports, case-series, studies for which the full text was unavailable, studies analyzing disinfectant solutions, and studies that did not involve materials intended for indirect restorations. No restrictions were applied regarding publication date or language, and no filters were used.

## Results

**Figure 1:**
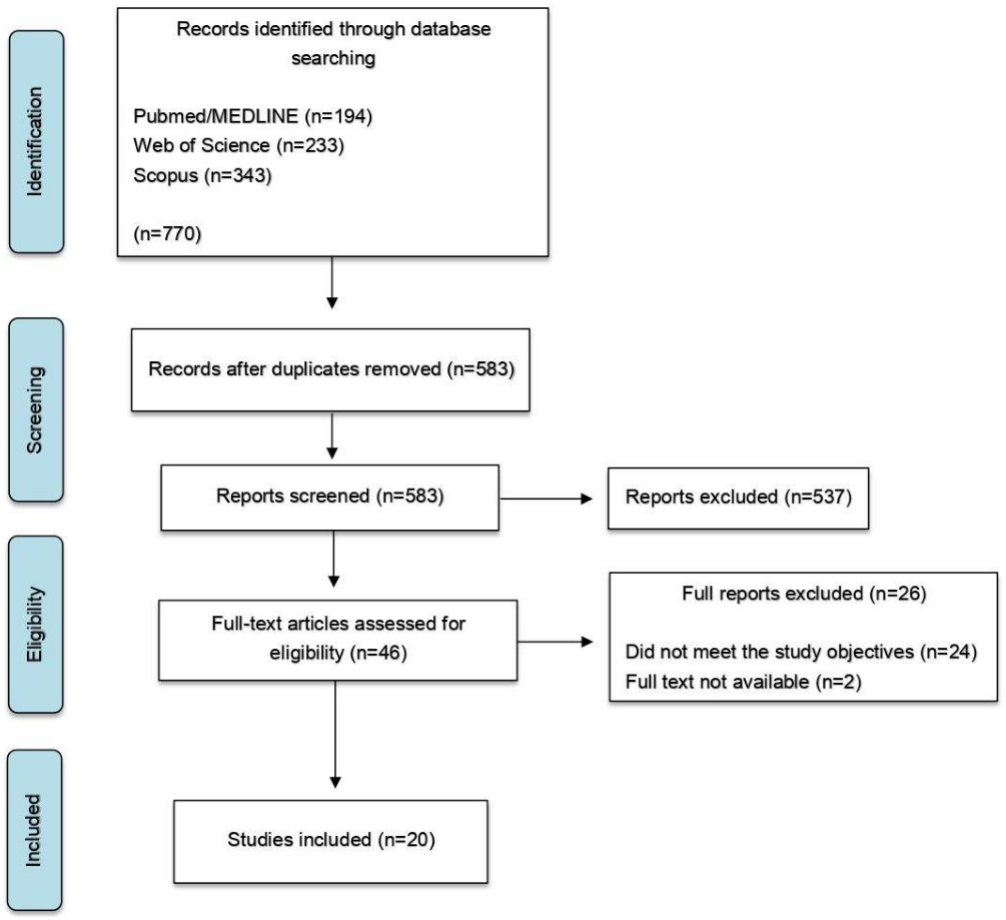
Flowchart of study selection.

Using the search strategy, a total of 770 articles were identified: 194 from PubMed/MEDLINE, 233 from Web of Science, and 343 from Scopus. After duplicate removal and screening of titles and abstracts, 46 articles were assessed through full-text reading, of which 20 met the eligibility criteria and were included, while 26 were excluded. The identification and inclusion process of studies from the electronic databases is illustrated in a flowchart (Fig. 1).

1

The characteristics of the included studies are summarized in Table 1.

Table 1

Among the 20 studies included, 3D-printed resins demonstrated a lower degree of microbial adhesion compared to other fabrication methods in six of them (1 , 13 , 19 - 22). However, four studies (2 , 23 - 25) reported unfavorable outcomes, and one study (26), found no statistically significant differences among the materials evaluated. Six studies (5 , 6 , 8 , 27 - 29) evaluated materials modified through the incorporation of different particles intended to enhance their microbiological properties. A reduced degree of microbial adhesion was observed in four of these studies (5 , 6 , 8 , 27). Conversely, two studies reported divergent results depending on the type of particle (28) and the alkyl chain length of the evaluated groups (29). The added particles included ZrO2 nanoparticles (6), graphene nanoplatelets (8), titanium dioxide and silanized chitosan nanoparticles (28), a synthesized fluoride complex (5), silver-loaded halloysite nanotubes (27), and quaternary ammonium compounds (29). Only one study (22) was conducted in vivo, while the remaining investigations were in vitro. Nine studies (2 , 5 , 10 , 13 , 20 , 21 , 24 , 28 , 30) reported the use of definitive 3D-printed resins for fixed prostheses. In contrast, ten studies (1 , 6 , 11 , 13 , 19 , 20 , 22 - 24 , 26) focused on 3D-printed resins intended for the fabrication of provisional crowns. Additionally, ten studies (2 , 5 , 10 , 13 , 19 , 20 , 25 , 26 , 28 , 29) used control groups in their analyses. Regarding the test materials, 14 studies (1 , 2 , 10 , 11 , 13 , 19 - 26 , 30), evaluated commercial resins aiming to compare their antimicrobial effects. Among these studies, additional aspects were investigated, such as different polishing protocols (21) and the influence of post-curing time and atmosphere on surface properties (30). Furthermore, two studies (11 , 24) analyzed surface finishing by polishing and glazing, confirming that post-processing significantly affects surface roughness and, consequently, microbial adhesion. Concerning polishing protocols, one study (24) used silicon carbide papers with grits of 1200, 2400, and 4000, while the other (11) used a micromotor with progressively finer prosthetic rubber abrasives. For the glazed groups, both studies coated the specimens with light-curable GC Optiglaze. With regard to specimen geometry, 15 studies (1 , 5 , 6 , 10 , 11 , 13 , 20 , 21 , 23 - 26 , 28 - 30) used disc-shaped specimens. In contrast, two studies (22 , 26) used dental crowns as test specimens, while four studies (2 , 19 , 26 , 27) used rectangular or cubic specimens to evaluate the level of microbial adhesion for each test material. Five studies (6 , 8 , 19 , 23 , 24) investigated the influence of artificial aging on the antimicrobial performance of the tested materials. Among them, three studies (6 , 8 , 19) demonstrated sustained effectiveness of the evaluated 3D-printed resins even after thermocycling, whereas two studies (23 , 24) reported greater effectiveness for milled resins.

## Discussion

The null hypothesis that 3D-printed restorative materials do not differ from those produced by other fabrication methods in terms of microbial adhesion was rejected, as material composition, printing parameters, and surface treatments demonstrated the potential to influence biofilm formation. When analyzing the adhesion of oral microorganisms to materials used for the fabrication of 3D-printed provisional crowns, the studies included in this review reported conflicting results. Several investigations observed that 3D-printed resins exhibited lower microbial adhesion than conventional materials, including bis-acrylics, (20) PMMA, (1) acrylic polymers, (13 , 19) bis-acrylics and composites, (19) as well as milled resins (1 , 13 , 19). Two of these studies (1 , 13) further evaluated commercial 3D-printed resins, both hybrid and temporary, and reported the lowest microbial adhesion indices for both categories when compared with milled resins. These findings have been attributed to favorable printing parameters, such as printing orientation and layer thickness, which may result in smoother surfaces (1). In contrast, other studies reported less favorable outcomes for 3D-printed resins, with increased surface roughness and biofilm formation compared with milled PMMA, conventional PMMA, and bis-acrylic resins. Such results were associated with the presence of surface grooves inherent to the layer-by-layer manufacturing process, as well as crack propagation caused by residual stresses arising from temperature variations during polymerization, which may create niches conducive to bacterial adhesion and proliferation (23 , 25). Regarding materials for the fabrication of permanent crowns by additive manufacturing, previous studies (6 , 8) have emphasized the reinforcement of the polymeric matrix with different types of fillers as a strategy to overcome mechanical strength limitations, thereby promoting the development of high-strength nanocomposites with improved longevity in the oral cavity. An in vitro study (9) investigated the microbiological effects of incorporating nanodiamonds into the resin matrix as reinforcing filler particles and demonstrated increased resistance to Streptococcus mutans biofilm formation. Nevertheless, within the oral environment, biofilm development is modulated by competitive interactions among diverse microbial species and by the presence of the salivary pellicle, which complicates a comprehensive assessment of material performance. Additionally, another study (10) evaluated microbial adhesion on resin-based hybrid ceramic materials and demonstrated that the chemical composition of the polymeric matrix and the initiator systems significantly influence surface roughness and biofilm formation. Consistently, an in vitro investigation using nanohybrid resins (11) showed that the incorporation of a polymerization inhibitor, associated with prolonged curing time, resulted in increased surface roughness. These findings suggest that specific formulation components may indirectly affect biological behavior by modifying surface characteristics that directly affects material-bacteria interactions. The gradual fusion between printing layers leads to increased porosity and the formation of deep grooves on the surface structure. Accordingly, some studies included in this review reported surface treatments aimed at achieving smoother surfaces and reducing microbial adhesion. One investigation (21) evaluated polishing protocols across different materials, including 3D-printed resins, and found that surface finishing significantly reduced bacterial adhesion, possibly due to the removal of unpolymerized resin residues from the specimen surfaces. In addition, horizontally printed methacrylate-based materials were shown to promote less biofilm formation than vertically printed specimens. Another study (11) assessed polished and glazed hybrid resin specimens as post-production treatments and observed lower microbial adhesion on glazed surfaces, whereas untreated specimens exhibited more pronounced biofilm formation, indicating that the additive manufacturing process itself does not inherently limit adhesion potential. Conversely, Kim et al. (24) reported that, despite glazing producing similar surface roughness and wettability among all tested resins, polished 3D-printed resins exhibited significantly higher roughness and microbial adhesion than polished milled resins. This difference was attributed to the surface characteristics of milled resins, which undergo pre-polymerization under high pressure and temperature, resulting in a more homogeneous structure. Furthermore, another study (30) investigated the influence of post-curing time and atmosphere on surface smoothness by assessing monomer conversion and demonstrated that surface roughness was significantly affected by resin type rather than by post-curing conditions, with glass-filler-reinforced specimens showing greater microbial adhesion. In contrast, another included study (1) emphasized that material selection should consider initial roughness parameters, as achieving surfaces resistant to microbial adhesion may require substantial investment and may still fail to ensure long-term durability due to the chemical and mechanical challenges of the oral environment. Consequently, further clinical studies are warranted to identify durable and clinically effective surface treatment strategies for reducing microbial adhesion. From another analytical perspective, studies investigating the incidence of microbial species on commercially available 3D-printed resins employed a wide diversity of microorganisms and distinct experimental combinations. One study (20), which used different microbial species, found that printed resins exhibited greater adhesion by representatives of the normal microbiota compared to fungal and periodontopathogenic species, consistent with the results of other studies included in this review (1 , 13). It has been argued that isolated periodontopathogenic species may demonstrate low adhesion due to the absence of primary bacterial species that mediate the initial attachment and promote the formation of a mixed polymicrobial biofilm on the substrate, underscoring the importance of evaluating the potential of each microbiota group when interpreting study outcomes (13). Conversely, other studies reported higher adhesion of Streptococcus mutans on printed resins compared to S. sanguinis (2 , 21) and Candida albicans (23). Several researchers highlight that compositional factors may influence microbial adhesion by modifying surface characteristics, which can lead to differences in colonization patterns among microorganisms. In this context, some studies indicate that S. mutans tends to exhibit greater adhesion to composite-based materials compared with other formulations (2 , 23). The fact that most studies reported in the literature and included in this review are in vitro constitutes a limitation, as the specific characteristics of the oral cavity require evaluation of materials under clinical conditions that better represent reality. Furthermore, factors such as the complex geometry of prostheses must be considered, since it is known that specimen shape influences the degree of microbial adhesion and surface characteristics (1). An in vitro study (26) used saliva from a single donor on specimens shaped as single crowns to simulate biofilm formation in a clinical environment, finding that 3D-printed resins did not differ significantly from conventionally fabricated acrylic and bis-acrylic resins. This outcome may be explained by the analysis being conducted at an early stage of biofilm development, which tends to change over time. Conversely, the in vivo study included in this review (22) found that 3D-printed provisional restorations exhibited lower colony counts of Streptococcus mutans, Streptococcus pyogenes, and Candida species compared to milled PMMA and heat-cured conventional PMMA. It is evident that, under clinical conditions, the salivary pellicle is a key determinant of microbial adhesion in the oral cavity, rendering surfaces more hydrophilic (9). Additionally, the presence of a polymicrobial biofilm and interspecies interactions represent unique aspects of clinical conditions, highlighting the need for further in vivo studies on this subject (13 , 21). The results of this review must be interpreted with caution. The studies exhibited methodological heterogeneity based on various factors, including differences in materials and intended applications, testing protocols, post-curing durations, artificial aging times, additional evaluated parameters, layer thicknesses, printing methods, and specimen geometries, thereby complicating high-precision comparisons. Furthermore, among the limitations, no risk of bias assessment tool was used in this review. However, this study compiles data that guide decision-making with direct implications for clinical practice. Its findings provide support for strategies that overcome inherent limitations of 3D printing, contributing to the reduction of microbial adhesion. Nonetheless further studies, particularly clinical evaluations, are essential to achieve a better understanding of the influence and variability in the performance of 3D-printed materials used in indirect restorations.

## Conclusions

The incorporation of nanoparticles, combined with appropriate technical parameters, enhances the antimicrobial efficacy of 3D-printed resins. Microbial adhesion is mainly influenced by material composition, printing parameters, and surface post-processing rather than by the printing technique alone. From a clinical perspective, optimizing layer thickness, printing orientation, and finishing protocols is essential to reduce surface irregularities and limit biofilm accumulation. Although most evidence is derived from in vitro studies, the findings suggest that properly optimized 3D-printed provisional restorations may present acceptable biological performance.

## Figures and Tables

**Table 1:** Synthesis of the studies included in this scoping review.

AUTHOR	STUDY TYPE	TESTED MATERIAL	MICROBIOLOGICAL ASPECTS ASSESSED	ADDITIONAL ASPECTS ASSESSED	CONCLUSION
Yue et al., 2015	In vitro	Conventional UDMA/GDMA-based resin matrix with the addition of quaternary ammonium compounds	Colony-forming unit count.	-	In quaternary ammonium chains with alkyl chain lengths of n = 8 and 12, significant differences in bacterial counts were observed compared to the control (p<0.01).
Liu et al., 2019	In vitro	Printed resin containing Ag-HNT	Microbial adhesion	-	The compound released Ag+ in artificial saliva and demonstrated sustained antimicrobial activity, without exhibiting cytotoxicity.
Arutyunov et al., 2020	In vitro	C&B resin (NextDent, Netherlands) and Freeprint Temp resin (Detax, Germany)	Colony-forming unit count.	-	Printed materials showed lower microbial adhesion compared to those fabricated with conventional methods (p<0.05).
Tsareva et al., 2020	In vitro	Milled resins: Temp Basic (Zirkonzahn, Italy), Re-Fine Acrylic (Yamahachi Dental, Japan);Printed resins: Freeprint Temp (Detax, Germany), C&B (NextDent, Netherlands), and Dental Sand (HARZ Labs, Russia)	Colony-forming unit count and Primary adhesion.	-	Significantly lower values were found for milled materials, and the lowest for printed materials compared with materials polymerized by cold and heat curing.	Aati, Shrestha et al., 2022	In vitro	C&B resin (NextDent, Netherlands) with the addition of ZrO₂ nanoparticles	Antimicrobial capacity and Biofilm formation	Biocompatibility and surface roughness	The addition of up to 5.0 wt% of ZrO₂ nanoparticles resulted in a significant antimicrobial effect (p≤ 0.05).
Aati, Chauhan et al., 2022	In vitro	C&B resin (NextDent, Netherlands) with the addition of graphene nanoplatelets	Antimicrobial activities	Surface roughness, topography, biocompatibility, flexural strength, microhardness, and nanoindentation	Graphene nanoplatelets provided an effective strategy for reducing C. albicans activity, with efficacy related to the concentration ratio.
Arutyunov et al., 2022	In vitro	Cold-cured resins: Belakril-M HO Tempo (LTD, Russia) and Luxatemp (DMG, Germany);Heat-cured resins: Belakril-M GO Tempo (LTD, Russia) and Sinma-M (AO, Ukraine);Milled resins: Temp Basic (Zirkonzahn, Italy) and Re-Fine (Yamahachi, Japan);Printed resins: FreePrint Temp (DETAX, Germany), C&B (NextDent, Netherlands), and Dental Sand (HARZ Labs, Russia)	Colony-forming unit count.	-	Printed materials exhibited the lowest initial microbial adhesion among the three tested microbiota types.
MazurekÃ¿Popczyk et al., 2022	In vitro	MFH resins (NextDent), 3D Plus (NextDent, Netherlands), and Mazic D Temp (Vericom, Korea)	Colony-forming unit count.	Surface roughness	Post-production processing led to reductions in surface roughness and biofilm parameters.
Simoneti; Pereira-Cenci; Dos Santos, 2022	In vitro	Stratasys SLS resin (selective laser sintering) and Gray Formlabs SLA resin (stereolithography)	Biofilm formation	Surface roughness, microhardness, flexural strength, fracture resistance, elastic modulus, and stress peak	No statistically significant differences were observed in biofilm formation (p > 0.05).
BÃ¤chle et al., 2023	In vitro	PEEK, PEKK, AKP, composite, PMMA, and 3D methacrylate	Microbial adhesion	Polishing protocols, surface roughness, and wettability	Significantly greater adhesion was observed in pressed PEEK compared to other manufacturing techniques, including milling, injection molding, and 3D printing (p < 0.001).
Ozer et al., 2023	In vitro	Printed resins: VarseoSmile Crown Plus (BEGO), Saremco Print Crown, and Formlabs 3D Permanent Crown	Colony-forming unit count	Surface roughness	The Saremco Crowntec group exhibited significantly higher bacterial adhesion than all other tested materials (p < 0.05). The lowest bacterial adhesion was recorded in the Cerasmart group (p < 0.05).
Parakaw et al., 2023	In vitro	Conventionally fabricated resin: Unifast Trad (GC, USA) and Protemp (3M ESPE, Germany);Milled resin: VIPIblock Trilux (VIPI, Brazil);Printed resin: C&B (NextDent, Netherlands)	Biofilm formation	Cytotoxicity, surface roughness, and gingival tissue attachment	P. gingivalis biofilm on Unifast Trad surfaces was significantly thicker than on VIPIblock Trilux (p ≤ 0.05) and the control (p ≤ 0.01), which showed the lowest values.
Ribeiro et al., 2023	In vitro	Bis-acrylic resin (Protemp 4, 3M ESPE, USA);Composite resin (Z350XT, 3M ESPE, USA);Milled resin (VIPI, SÃ£o Paulo, Brazil);Printed resin (Cosmos Temp, Yller, Brazil)	Colony-forming unit count	Aging, surface roughness, porosity, and flexural strength	No statistical differences were observed among the groups, all of which showed low microbial adhesion.
Taşın; Güvenir; Ismatullaev, 2023	In vitro	Conventional technique resin: A-PMMA Imident (Imicryl Dental, Türkiye) and PreVISION Temp bis-acrylate (Kulzer GmbH, Germany);Milled technique: PMMA Disc Multi (Sagemax Bioceramics, USA);Printed composite resin: Temporis (DWS, Italy)	Adhesion of Streptococcus mutans and Candida albicans assessed by colony-forming unit counts	Surface roughness and wettability	S. mutans adhesion was significantly higher in the bis-acrylate and printed groups, whereas C. albicans adhesion was significantly greater in the autopolymerized PMMA and printed groups (p = 0.001).
ElMalah et al., 2024	In vitro	VarseoSmile Crown Plus resins (BEGO) containing TiO2 NPs and sCS NPs	Microbial adhesion	Flexural strength and color stability	The incorporation of TiO2 NPs led to a reduction in S. mutans compared to the chitosan group, thereby enhancing its antimicrobial efficacy (p=0.007).
Jin et al., 2024	In vitro	UA-based resin with the addition of a fluoride complex (Sigma Aldrich, USA)	Colony-forming unit count	Hardness, flexural strength, and fluoride release	S. mutans colony counts were significantly lower in the 10F-UA group compared with the 5F-UA group (p < 0.05) and compared to the reference and control materials.
Kim et al., 2024	In vitro	TC-80DP resin (Graphy, Korea) and glass filler-reinforced composite resin (Crowntec, Saremco, Switzerland)	Colony-forming unit count and Protein adsorption.	PPT (standard or extended) and PPA (air or nitrogen), surface roughness, wettability, and cytotoxicity	The resin with glass fillers showed significantly higher microbial adhesion compared to the TC-80DP resin group (p < 0.001).
Kim et al., 2024	In vitro	Permanent Crown resin (Formlabs), Temporary CB resin (Formlabs), and 2M2 resin (Vita Enamic)	Colony-forming unit count and Protein adsorption	Material type and surface finishing (polished or glazed), wettability, and surface roughness	The printed resin exhibited greater microbial retention under both polished and glazed conditions compared to the Temporary resin and the milled resin (p < 0.001).
Kumari et al., 2024	In vivo	Heat-cured PMMA, milled PMMA, and printed oligomers	Colony-forming unit count.		Crowns fabricated with heat-curing exhibited the highest colony-forming unit (CFU) counts, followed by milled crowns and 3D-printed crowns (p< 0.05).
Wang et al., 2024	In vitro	Hybrid resin–ceramic materials: Crowntec (Saremco, Switzerland), VarseoSmile Crown Plus (Bego, Germany), Tera Harz TC-80DP (Graphy, South Korea), C&B Permanent (ODS, South Korea), Formlabs Permanent Crown (Formlabs, USA), and HeyGears (China).	Colony-forming unit count (CFU count)	Surface roughness, wettability, and cytotoxicity	The C&B Permanent group presented the highest colony-forming unit count, whereas the Tera Harz TC-80DP group exhibited the lowest number of colonies (P < 0.05).

1

## Data Availability

The datasets used and/or analyzed during the current study are available from the corresponding author.
